# Supplementary material for: Interdisciplinary optimism? Sentiment analysis of Twitter data
Source: R Soc Open Sci. 2019 Jul 31;6(7):190473. doi: 10.1098/rsos.190473 (PMC6689644; doi:10.1098/rsos.190473)
Supplement: Supplements S3 [file rsos190473supp3.pdf]

Text S1 Preprocessing Tweets

Tweeting, the process of publishing a tweet, proceeds in the form of free text, often in combination with special characters, symbols, emoticons, and emoji. This, in combination with a character limit, make tweeters creative and concise in their writing, favoring brevity over readability to convey their message—even more so with the 140 characters limit. Thus tweet data is highly idiosyncratic and several preprocessing steps were necessary (described below) to make the dataset suitable for sentiment analysis.

Retweets and duplicate tweets We removed retweets, identified by the string 'RT' preceding the tweet, as they essentially are duplicates of the initial or first tweet. Additionally, duplicate tweets that were identical in their content were also excluded.

Non-English tweets We focused our analysis on English tweets only and excluded all non-English tweets according to the 'lang' attribute provided by the Twitter API.

User tags and URLs For the purpose of sentiment analysis, the user tags (i.e., mentioning of other Twitter user accounts by using @) and URLs (i.e., a link to a specific website) convey no specific sentiment and were therefore replaced with a suitable placeholder (e.g. `USER`, `URL`). As a result, the presence and frequency of user tags and URLs were retained and normalized.

Hashtags Hashtags are an important element of Twitter and can be used to facilitate a search while simultaneously convey opinions or sentiments. For example, the hashtag #love reveals a positive sentiment or feeling, and tweets using the hashtag are all indexed by #love. Twitter allows users to create their own hashtags and poses no restrictions in appending the hashtag symbol (i.e., #) in front of any given text. Following the example of the #love hashtag, we preprocessed hashtags by removing the hash sign, essentially making #love equal to the word *love*.

Contractions and repeating characters Contractions, such as *don't* and *can't*, are a common phenomenon in the English spoken language and, generally, less common in formal written text. For tweets, contractions can be found in abundance and are an accepted means of communication. Contractions were preprocessed by splitting them into their full two-word expressions, such as *do not* and *can not*. In doing so, we normalized contractions with their "decontracted" counterparts. Another phenomenon occurring in tweets is the use of repeating characters, such as *I loveeeee it*, often used for added emphasis. Words that have repeated characters are limited to a maximum of two consecutive characters. For example, the word *loveee* and *loveeee* are normalized to *lovee*. In doing so, we maintained some degree of emphasis.

Lemmatization and uppercase words For grammatical reasons, different word forms or derivationally related words can have a similar meaning and, ideally, we would want such terms to be grouped together. For example, the words *like*, *likes*, and *liked* all have similar semantic meaning and should, ideally, be normalized. Stemming and lemmatization are two NLP techniques to reduce inflectional and derivational forms of words to a common base form. Stemming heuristically cuts off derivational affixes to achieve some kind of normalization, albeit crude in most cases. We applied

lemmatization, a more sophisticated normalization method that uses a vocabulary and morphological analysis to reduce words to their base form, called lemma. It is best described by its most basic example, normalizing the verbs *am*, *are*, *is* to *be*, although such terms are not important for the purpose of sentiment analysis. Additionally, uppercase and lowercase words were grouped as well.

Emoticons and Emojis Emoticons are textual portrayals of a writer's mood or facial expressions, such as :-) and :-D (i.e., smiley face). For sentiment analysis, they are crucial in determining the sentiment of a tweet and should be retained within the analysis. Emoticons that convey a positive sentiment, such as :-), :-], or ;), were replaced with the positive placeholder word `EM_POS`; in essence, grouping variations of positive emoticons with a common word. Emoticons conveying a negative sentiment, such as :-(, :c, or :-c, were replaced by the negative placeholder word `EM_NEG`. A total of 47 different variations of positive and negative emoticons were replaced. A similar approach was performed with emojis that resemble a facial expression and convey a positive or negative sentiment. Emojis are graphical symbols that can represent an idea, concept or mood expression, such as the graphical icon of a happy face. A total of 40 emojis with positive and negative facial expressions were replaced by the placeholder word `EM_POS` and `EM_NEG`, respectively. Replacing and grouping the positive and negative emoticons and emojis will result in the sentiment classification algorithm learning an appropriate weight factor for the corresponding sentiment class. For example, tweets that have been labeled as conveying a negative sentiment (by a human annotator for instance) and predominantly containing negative emoticons (e.g., :-(), can result in the classification algorithm assigning a higher probability or weight to the negative sentiment class for such emoticons. Note that this only holds when the neutral and positively labeled tweets do not predominantly contain negative emoticons; otherwise their is no discriminatory power behind them.

Numbers, punctuation, and slang Numbers and punctuation symbols were removed, as they typically convey no specific sentiment. Numbers that were used to replace characters or syllables of words were retained, such in the case of *see you l8er*. We chose not to convert slang and abbreviations to their full word expressions, such as *brb* for *be right back* or *ICYMI* for *in case you missed it.* The machine learning model, described later, would correctly handle most common uses of slang, with the condition that they are part of the training data. As a result, slang that is indicative of a specific sentiment class (e.g. positive or negative) would be assigned appropriate weights or probabilities during model creation.

Input features Each tweet was tokenized, the process of obtaining individual words from sentences. Furthermore, we represented tweets as count vectors with and without inverse document frequency (IDF) weighting [1]. Different variations of tokenization were explored, such as 1-word (unigram), 2-word (bigrams), 3-word (trigrams), and 4-word (n-gram) combinations. Bi-grams are especially important to capture negation of words combinations, such as *not good* or *not great*, that would not be captured when using 1-word (unigram) features alone.

Text S2 Descriptions of the seven datasets used to train the sentiment classifier

Sanders The Sanders dataset consists out of 5,513 hand classified tweets related to the topics Apple (@Apple), Google (#Google), Microsoft (#Microsoft), and Twitter (#Twitter). Tweets were classified as positive, neutral, negative, or irrelevant; the latter referring to non-English tweets which we discarded. The Sanders dataset has been used for boosting Twitter sentiment classification using different sentiment dimensions [2], combining automatically and hand-labeled twitter sentiment labels [3], and combining community detection and sentiment analysis [4]. The dataset is available from `http://www.sananalytics.com/lab/`.

Obama-McCain Debate (OMD) The Obama-McCain Debate (OMD) dataset contains 3,238 tweets collected in September 2008 during the United States presidential debates between Barack Obama and John McCain. The tweets were collected by querying the Twitter API for the hash tags #tweetdebate, #current, and #debate08 [5, 6]. A minimum of three independent annotators rated the tweets as positive, negative, mixed, or other. Mixed tweets captured both negative and positive components. Other tweets contained non-evaluative statements or questions. We only included the positive and negative tweets with at least two-thirds agreement between annotators ratings; mixed and other tweets were discarded. The OMD dataset has been used for sentiment classification by social relations [7], polarity classification [8], and sentiment classification utilizing semantic concept features [9]. The dataset is available from `https://bitbucket.org/speriosu/updown`.

Stanford Test The Stanford Test dataset contains 182 positive, 139 neutral, and 177 negative annotated tweets [10]. The tweets were labeled by a human annotator and were retrieved by querying the Twitter search API with randomly chosen queries related to consumer products, company names and people. The Stanford Training dataset, in contrast to the Stanford Test dataset, contains 1.6 million labeled tweets. However, the 1.6 million tweets were automatically labeled, thus without a human annotator, by looking at the presence of emoticons. For example, tweets that contained the positive emoticon :-) would be assigned a positive label, regardless of the remaining content of the tweet. Similarly, tweets that contained the negative emoticon :-(would be assigned a negative label. Such an approach is highly biased [11] and we choose not to include this dataset for the purpose of creating a sentiment classifier from labeled tweets. The Stanford Test dataset, although relatively small, has been used to analyze and represent the semantic content of a sentence for purposes of classification or generation [12], semantic smoothing to alleviate data sparseness problem for sentiment analysis [13], and sentiment detection of biased and noisy tweets [14]. The dataset is available from `http://www.sentiment140.com/`.

Health Care Reform (HCR) The Health Care Reform (HCR) dataset was created in 2010 – around the time the health care bill was signed in the United States – by extracting tweets with the hashtag #hcr [8]. The tweets were manually annotated by the authors by assigning the labels positive, negative, neutral, unsure, or irrelevant. The dataset was split into training, development and test data. We combined the three different datasets that contained a total of 537 positive, 337 neutral, and 886 negative tweets. The tweets labeled as irrelevant or unsure were not included. The HCR dataset was used to improve sentiment analysis by adding semantic features to tweets [9]. The dataset is available from `https://bitbucket.org/speriosu/updown`.

SemEval-2016 The Semantic Analysis in Twitter Task 2016 dataset, also known as SemEval-2016 Task 4, was created for various sentiment classification tasks. The tasks can be seen as challenges where teams can compete amongst a number of sub-tasks, such as classifying tweets into positive, negative and neutral sentiment, or estimating distributions of sentiment classes. Typically, teams with better classification accuracy or other performance measure rank higher. The dataset consist of training, development, and development-test data that combined consist of 3,918 positive, 2,736 neutral, and 1,208 negative tweets. The original dataset contained a total of 10,000 tweets − 100 tweets from 100 topics. Each tweet was labeled by 5 human annotators and only tweets for which 3 out of 5 annotators agreed on their sentiment label were considered. For a full description of the dataset and annotation process see [15]. The dataset is available from `http://alt.qcri.org/semeval2016/task4/`.

Sentiment Strength (SS) The Sentiment Strength (SS) dataset was used to detect the strength of sentiments expressed in social web texts, such as tweets, for the sentiment strength detection program SentiStrength [16]. The dataset was labeled by human annotators and each tweet was rated on a scale from 1 to 5 for both positive and negative sentiment, i.e. a dual positive-negative scale. For the purpose of this paper, we re-labeled the tweets into positive, negative and neutral tweets as follows. Tweets were considered positive if the positive score was at least 1.5 times larger than the negative score; a positive score of 4 and a negative score of 1 would result in a positive label. Tweets that have a negative score of 1.5 times larger than the positive score were considered negative. A similar score on the positive and negative scale would result in a neutral tweet, such when the positive score is 2 and the negative score 2. A similar re-labeling process was performed by [11]. A total of 1,252 positive, 1,952 neutral, and 861 negative tweets were used. SentiStrength has been used to quantify and statistically validate trading assets from social media data [17], and analyzing emotional expressions and social norms in online chat communities [18]. The dataset is available from `http://sentistrength.wlv.ac.uk/documentation/`

CLARIN 13-Languages The CLARIN 13-languages dataset contains a total of 1.6 million labeled tweets from 13 different languages, the largest sentiment corpus made publicly available [19]. We used the English subset of the dataset since we restricted our analysis to English tweets. Tweets were collected in September 2013 by using the Twitter Streaming API to obtain a random sample of 1% of all publicly available tweets. The tweets were manually annotated by assigning a positive, neutral, or negative label by a total of 9 annotators; some tweets were labeled by more than 1 annotator or twice by the same annotator. For tweets with multiple annotations, only those with two-third agreement were kept. The original English dataset contained around 90,000 labeled tweets. After recollection, a total of 15,064 positive, 24,263 neutral, and 12,936 negative tweets were obtained. The dataset is available from `http://hdl.handle.net/11356/1054`.

Figures

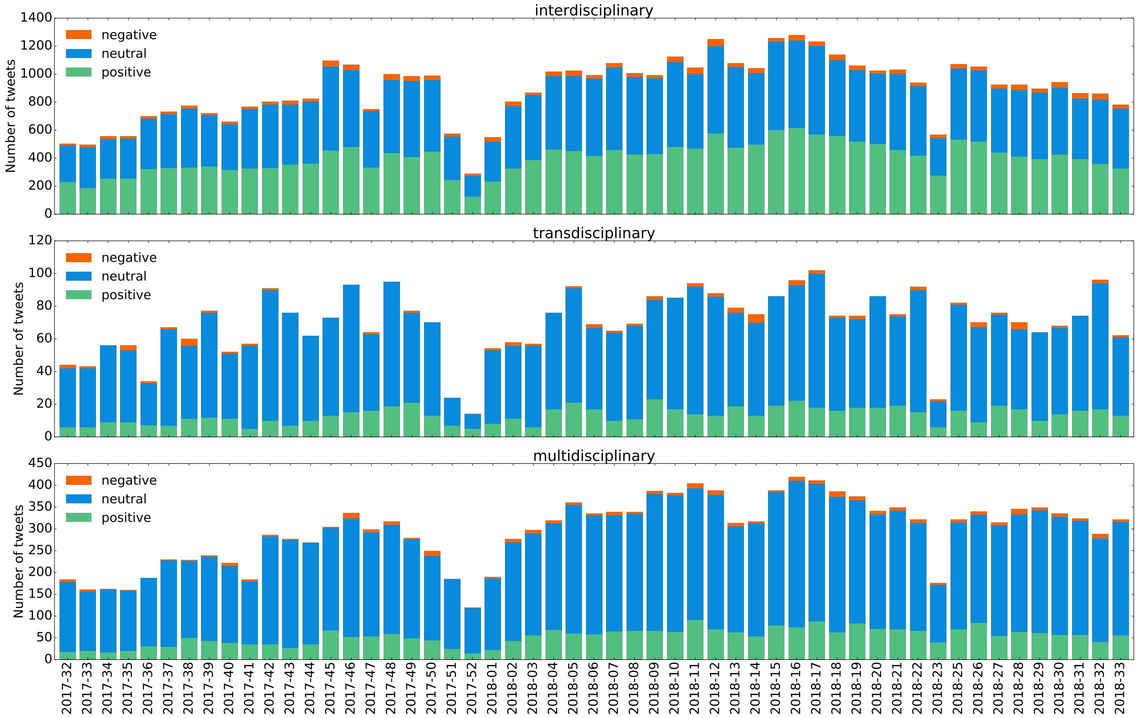

Figure S1: Sentiment over time

Tables

Table S1: Overview of explored hyper-parameter values when performing a cross-validated grid search to obtain the machine learning classification model with best classification performance (F1-score). The value 'x' indicates that the hyper-parameter was used to explore different variations of the algorithm. Not all hyper-parameters are possible for all explored models, these are indicated by the absence of an 'x'. A full description of the hyper-parameters can be found in the Scikit-learn documentation at https://scikit-learn.org/stable/documentation.html

**Hyper-parameter**	**Value Range**	**SVM**	**Logisitic Regression**	**Multinomial NB**	**Bernoulli NB**	**Descision Trees**	**ADA Boost**	**ML-Perceptron**
n-grams	1–4	x	x	x	x	x	x	x
min-df	0,5,10,15	x	x	x	x	x	x	x
max-df	1.0, 0.95, 0.90	x	x	x	x	x	x	x
IDF	Yes, No	x	x	x	x	x	x	x
sublinear-TF	Yes, No	x	x	x	x	x	x	x
C (penalty term)	0.001, 0.01, 0.5, 0.1, 1, 5, 10, 15, 20, 100	x	x					
fit prior	Yes, No			x	x			
alpha	0.001, 0.01, 0.5, 0.1, 1, 5, 10, 15, 20, 100			x	x			
splitter	best, random					x		
criterion	gini, entropy					x		
max features	auto, sqrt, log2, None					x		
num. estimators	100,200,300,400,500						x	
algorithm	SAMME, SAMME.R						x	
neural architectures	(100,50,20),(200, 100, 100),(300, 50, 50, 50),(50, 40, 30, 10),(20, 30, 50, 50),(70, 50, 40, 30)						x	
Cross-validation	10-Fold	x	x	x	x	x	x	x
	Total Models (x1000)	19.2	19.2	38.4	38.4	30.7	19.2	11.5

Table S2: Hyper-parameter values that resulted in highest F1 score for the seven explored classification algorithms.

**Hyper-parameter**	**SVM**	**Logisitic Regression**	**Multinomial NB**	**Bernoulli NB**	**Descision Trees**	**ADA Boost**	**ML-Perceptron**
n-grams	1–4	1–4	1–3	1–2	1–4	1–4	1–3
min-df	0	0	5	5	5	5	5
max-df	1	1	1	1	1	1	1
IDF	no	no	n	yes	no	no	no
sublinear-TF	yes	yes	yes	yes	yes	yes	yes
C	0.5	10					
fit-prior			yes	no			
alpha			0.1	1			
splitter					random		
criterion					gini		
max-features					None		
n-estimator						400	
algorithm						SAMME.R	
neural architecture							200,100,100

References

[1] Salton G, McGill MJ. 1982 *Introduction to Modern Information Retrieval.* New York, NY, USA: McGraw-Hill, Inc.

[2] Bravo-Marquez F, Mendoza M, Poblete B. 2013 Combining strengths, emotions and polarities for boosting Twitter sentiment analysis. In *Proceedings of the Second International Workshop on Issues of Sentiment Discovery and Opinion Mining - WISDOM '13* pp. 1–9 New York, NY, USA. ACM Press.

[3] Liu KL, Li WJ, Guo M. 2012 Emoticon Smoothed Language Models for Twitter Sentiment Analysis. In *Proceedings of the Twenty-Sixth AAAI Conference on Artificial Intelligence* pp. 1678–1684 Toronto, Ontario, Canada. AAAI Press.

[4] Deitrick W, Hu W. 2013 Mutually Enhancing Community Detection and Sentiment Analysis on Twitter Networks. *Journal of Data Analysis and Information Processing* **01**, 19–29.

[5] Shamma Da, Kennedy L, Churchill EF. 2009 Tweet the debates. In *Proceedings of the first SIGMM workshop on Social media* pp. 3–10 Beijing, China. ACM Press.

[6] Diakopoulos NA, Shamma DA. 2010 Characterizing debate performance via aggregated twitter sentiment. In *Proceedings of the SIGCHI Conference on Human Factors in Computing Systems* pp. 1195–1198 Atlanta, Georgia, USA. ACM Press.

[7] Hu X, Tang L, Tang J, Liu H. 2013 Exploiting social relations for sentiment analysis in microblogging. In *Proceedings of the sixth ACM international conference on Web search and data mining - WSDM '13* pp. 537–546 New York, New York, USA. ACM Press.

[8] Speriosu M, Sudan N, Upadhyay S, Baldridge J. 2011 Twitter Polarity Classification with Label Propagation over Lexical Links and the Follower Graph. In *Proceedings of the Conference on Empirical Methods in Natural Language Processing* pp. 53–56 Stroudsburg, PA, USA. Association for Computational Linguistics.

[9] Saif H, He Y, Alani H. 2012 Semantic Sentiment Analysis of Twitter. In *Proceedings of the 11th international conference on The Semantic Web* vol. 1 pp. 508–524 Berlin, Heidelberg. Springer-Verlag.

[10] Go A, Bhayani R, Huang L. 2009 Twitter Sentiment Classification using Distant Supervision. Technical report CS224N Project Report, Stanford.

[11] Saif H, Fernandez M, He Y, Alani H. 2013 Evaluation datasets for Twitter sentiment analysis a survey and a new dataset, the STS-Gold. In *Proceedings of the 1st Workshop on Emotion and Sentiment in Social and Expressive Media (ESSEM)* vol. 1096 pp. 9–21 Turin, Italy. CEUR.

[12] Kalchbrenner N, Grefenstette E, Blunsom P. 2014 A Convolutional Neural Network for Modelling Sentences. In *Proceedings of the 52nd Annual Meeting of the Association for Computational Linguistics (Volume 1: Long Papers)* pp. 655–665 Stroudsburg, PA, USA. Association for Computational Linguistics.

[13] Saif H, He Y, Alani H. 2011 Semantic smoothing for Twitter sentiment analysis. In *Proceeding of the 10th International Semantic Web Conference (ISWC)* pp. 23–27 Bonn, Germany. Springer.

[14] Barbosa L, Feng J. 2010 Robust Sentiment Detection on Twitter from Biased and Noisy Data. In *Proceedings of the 23rd International Conference on Computational Linguistics* pp. 36–44 Beijing, China. Association for Computational Linguistics.

[15] Nakov P, Ritter A, Rosenthal S, Stoyanov V, Sebastiani F. 2016 SemEval-2016 Task 4: Sentiment Analysis in Twitter. In *Proceedings of the 10th International Workshop on Semantic Evaluation* pp. 1–18 San Diego, California. Association for Computational Linguistics.

[16] Thelwall M. 2017 The Heart and Soul of the Web? Sentiment Strength Detection in the Social Web with SentiStrength. In Holyst J, editor, *Cyberemotions. Understanding Complex Systems* , pp. 119–134. Switzerland: Springer International Publishing.

[17] Zheludev I, Smith R, Aste T. 2015 When Can Social Media Lead Financial Markets?. *Scientific Reports* **4**, 4213.

[18] Garas A, Garcia D, Skowron M, Schweitzer F. 2012 Emotional persistence in online chatting communities. *Scientific Reports* **2**, 402.

[19] Mozetič I, Grčar M, Smailović J. 2016 Multilingual Twitter Sentiment Classification: The Role of Human Annotators. *PLOS ONE* **11**, e0155036.
